# Controlling Crystallization of Aqueous-Processed Planar Perovskite Films via Sodium Dodecyl Sulfonate Surfactant Modulation

**DOI:** 10.3390/molecules30102146

**Published:** 2025-05-13

**Authors:** Na Zheng, Cunyun Xu, Xiaofeng He, Gaobo Xu, Jiancheng You, Zhongjun Dai, Han Jiang, Qianqian Zhang, Qunliang Song

**Affiliations:** Institute for Clean Energy and Advanced Materials, School of Materials and Energy, Southwest University, Chongqing 400715, China; zn1981608813@email.swu.edu.cn (N.Z.); cyxuamos@foxmail.com (C.X.); hexf1992@163.com (X.H.); gbxu1805@163.com (G.X.); youjiancheng2021@163.com (J.Y.); jlovendai@163.com (Z.D.); 18283003178@163.com (H.J.); 18311651723@163.com (Q.Z.)

**Keywords:** water, aqueous lead nitrate, planar inverted perovskite solar cells, surfactant, crystallization optimization

## Abstract

Solution processing represents a widely adopted methodology for perovskite solar cell (PSC) fabrication. Nevertheless, the prevalent use of toxic solvents and anti-solvents in conventional approaches presents significant challenges for PSC commercialization. Water, as an environmentally benign solvent with exceptional Pb(NO_3_)_2_ solubility, offers a promising alternative for perovskite film preparation. However, the sluggish conversion kinetics of Pb(NO_3_)_2_ to perovskite often results in morphological imperfections and incomplete conversion, particularly detrimental to planar inverted PSCs derived from aqueous solutions, which currently exhibit limited power conversion efficiencies (PCE) of approximately 6%. To mitigate the Ostwald ripening effect induced by slow reaction kinetics and enhance the conversion efficiency of deep-layer Pb(NO_3_)_2_ and PbI_2_, this study proposes a strategy of increasing the pore size in porous Pb(NO_3_)_2_ structures. Through the incorporation of sodium dodecyl sulfonate (SDS) surfactant into the Pb(NO_3_)_2_ precursor solution, we successfully fabricated high-quality perovskite films. Comprehensive characterization revealed that SDS doping effectively modified the surface properties of Pb(NO_3_)_2_ films, accelerating their conversion to perovskite. The optimized PSCs based on SDS-modified perovskite films demonstrated improved energy level alignment, enhanced charge carrier extraction, and suppressed non-radiative recombination. Consequently, the PCE of planar inverted aqueous PSCs increased significantly from 12.27% (control devices) to 14.82% following surfactant modification. After being stored in a nitrogen glove box for 800 h, the performance of the device still remained above 90% of its original level. It can still maintain 60% of its original performance after a 100 h heating aging test at 80 degrees.

## 1. Introduction

Perovskite solar cells (PSCs) have emerged as a prominent photovoltaic technology in recent years owing to their exceptional optoelectronic properties, including high absorption coefficients, superior charge carrier mobility, extended diffusion lengths, and tunable bandgaps [1,2,3]. Remarkable progress has been evidenced by the dramatic efficiency enhancement from an initial 3.8% to a current certified record of 26.7%, underscoring their commercial viability. Solution processing remains the predominant fabrication approach for PSCs due to its simplicity and scalability. Conventional one-step solution methods typically employ organic solvents such as dimethyl sulfoxide (DMSO) [4], N,N-dimethylformamide (DMF) [5], N-methylpyrrolidone (NMP) [6], and γ-butyrolactone (GBL) [7] to dissolve organic ammonium salts and metal halides. Anti-solvents, including chlorobenzene (CB), toluene (Tol), and diethyl ether (DEE), are subsequently utilized to enhance film quality. However, these solvents pose significant health risks, particularly to hepatic, respiratory, and neurological systems [8], leading to stringent regulatory restrictions in various countries.

While alternative solvents with reduced toxicity, such as ethyl acetate, triethyl phosphate, methylammonium formate, and potassium ammonium acetate, have been explored as green alternatives [9,10], their large-scale industrial application remains problematic. From an environmental perspective, water represents an ideal solvent candidate, offering both cost-effectiveness and non-toxicity. Nevertheless, challenges persist due to the limited solubility of lead halides in aqueous systems and the inherent susceptibility of perovskite films to moisture-induced degradation [11].

The pioneering work by Wei et al. demonstrated the feasibility of aqueous-based PSC fabrication using Pb(NO_3_)_2_ precursors through the sequential deposition method, achieving a relatively low power conversion efficiency (PCE) of 12.58% [12]. Despite this advancement, the conversion kinetics from Pb(NO_3_)_2_ to perovskite remains sluggish [13,14,15], compounded by the detrimental effects of nitrate ions on newly formed metastable perovskite [16]. Recent efforts have focused on enhancing the conversion efficiency of Pb(NO_3_)_2_ and mitigating the degradation of perovskite, including: (1) Process optimization: Muhammad Adnan developed a multi-soaking annealing protocol to improve Pb(NO_3_)_2_ conversion and film quality. (2) Deposition techniques: Yue Feng demonstrated enhanced conversion rates through multiple drop-casting approaches [17]. (3) Crystallinity control: Zhai et al. achieved pinhole-free, large-grain films via dry air and oleate anion-mediated crystallization [18]. (4) Nucleation engineering: Nano-seed fluids have been employed to reduce nucleation barriers and refine grain structures [19]. (5) Photon-assisted synthesis: Light irradiation has been utilized to modulate the PbI_2_ microstructure and facilitate lead chloride conversion [20]. These advancements have propelled the PCE of aqueous-processed PSCs to 24%, comparable to the performance of organic solvent-based counterparts.

Previous research on high-performance PSCs utilizing aqueous Pb(NO_3_)_2_ precursors has predominantly focused on mesoporous TiO_2_-based regular (n-i-p) architectures. However, these configurations present several limitations: (1) the necessity for high-temperature sintering of mesoporous materials increases energy consumption and fabrication complexity, and (2) the reliance on costly spiro-OMeTAD as the hole transport layer. In contrast, planar inverted (p-i-n) PSCs offer distinct advantages, including low-temperature processing compatibility, enhanced flexibility, cost-effectiveness, and simplified fabrication procedures [21]. Despite these benefits, planar inverted PSCs fabricated from aqueous Pb(NO_3_)_2_ precursors have demonstrated limited efficiencies, reaching only 6.46% [22].

The fundamental challenge in Pb(NO_3_)_2_-based perovskite formation lies in the complex transformation mechanism, which involves MAX solution infiltration and substantial volume changes. Particularly noteworthy is the approximately two-fold volumetric expansion during the PbI_2_-to-perovskite conversion [23]. This transformation process leads to surface pore blockage in the Pb(NO_3_)_2_ layer, impeding MAX solution infiltration and hindering the conversion of underlying Pb(NO_3_)_2_ and PbI_2_. Furthermore, the inherently slow conversion kinetics in the Pb(NO_3_)_2_/H_2_O system exacerbates these issues through Ostwald ripening effects [20], which promote crystal growth at the expense of smaller particles, thereby intensifying the pore clogging phenomenon.

To address these challenges, strategic enlargement of the Pb(NO_3_)_2_ film’s pore structure emerges as a promising solution. This approach facilitates deeper MAX solution penetration, mirroring the successful implementation of porous architectures in regular PSCs that have enabled high efficiencies in aqueous Pb(NO_3_)_2_-based systems. Addressing the incomplete conversion issue in aqueous Pb(NO_3_)_2_-based planar perovskite films remains an urgent challenge. In this study, we employ sodium dodecyl sulfonate (SDS) as a surfactant additive, building upon previous literature reports [24,25], to systematically investigate its impact on the Pb(NO_3_)_2_-to-perovskite transformation. Our experimental results reveal that SDS modification significantly enhances the conversion process through multiple mechanisms: (1) modification of the Pb(NO_3_)_2_ film surface properties, (2) accelerated MAX solution diffusion and subsequent Pb(NO_3_)_2_ conversion, and (3) improved energy level alignment between the hole transport layer and perovskite.

The incorporation of SDS enables the fabrication of uniform, dense perovskite films with reduced structural defects. Notably, the sulfonic acid groups in SDS effectively coordinate with under-coordinated Pb^2+^ ions, leading to substantial suppression of non-radiative recombination. Furthermore, the SDS-modified devices demonstrate enhanced charge carrier extraction and reduced lattice strain during the conversion process. These synergistic effects result in a remarkable improvement in device performance, with champion planar inverted PSCs achieving a power conversion efficiency of 14.82%—a significant enhancement compared to the 12.27% efficiency of reference devices without SDS additive.

## 2. Results and Discussion

Figure S1 shows a molecular structure diagram of SDS, and Figure S2 presents the schematic flowchart of the preparation of perovskite films from Pb(NO_3_)_2_ with and without SDS doping. As indicated in the flowchart, different concentrations of SDS aqueous solutions were added into the Pb(NO_3_)_2_ solution to finally form mixed solutions with a concentration of 1.8 M of Pb(NO_3_)_2_ and different concentrations (0, 1 × 10^−2^, 1.6 × 10^−5^, 6.4 × 10^−7^ mg·mL^−1^) of SDS. Then, their impacts on the device performance were investigated by evaluating the performance of the PSC devices. The PSCs were obtained by a two-step method: a Pb(NO_3_)_2_ film was prepared by spin coating aqueous Pb(NO_3_)_2_ precursor solution, and then perovskite was slowly formed in an organic mixed halide (MAX) solution containing MACl and MAI. By comparing the perovskite film without SDS (control device), 1.6 × 10^−5^ mg·mL^−1^ was found out to be the optimal doping concentration.

According to literature reports, the conversion from Pb(NO_3_)_2_ to perovskite film can be divided into two sequential steps: Pb(NO_3_)_2_ first reacts rapidly with MAX to generate PbI_2_, and then PbI_2_ slowly converts into perovskite within hundreds of seconds.

Pb(NO_3_)_2_ + MAI → PbI_2_ + MANO_3_ (1)

PbI_2_ + MAI ↔ MAPbI_3_ (2)

**Figure 1:**
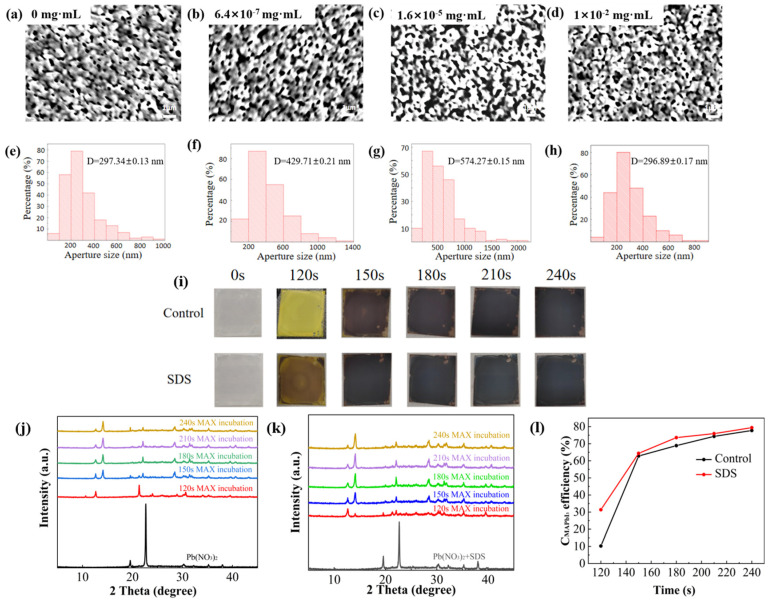
(**a**–**d**) SEM images corresponding to different concentrations (0, 6.4 × 10^−7^, 1.6 × 10^−5^, 1 × 10^−2^ mg·mL^−1^) of SDS doping. (**e**–**h**) Statistical charts of the corresponding pore sizes based on the SEM images with different concentrations (0, 6.4 × 10^−7^, 1.6 × 10^−5^, 1 × 10^−2^ mg·mL^−1^) of SDS doping. (**i**) Digital photo comparison after different immersion durations. XRD patterns of the (**j**) control and (**k**) SDS-doped groups after different immersion durations. (**l**) Conversion rates of MAPbI_3_ at different immersion times.

The slow transformation in the latter stage easily induces the well-known Ostwald ripening effect, and volume expansion occurs during the conversion from PbI_2_ to perovskite. Therefore, the state of the Pb(NO_3_)_2_ film directly affects the infiltration of the MAX solution and the conversion rate. As SEM shows in Figure 1a–d, Pb(NO_3_)_2_ films treated with SDS do show some morphology changes. The estimations of pore sizes of the Pb(NO_3_)_2_ films are shown in Figure 1e–h, demonstrating the largest pore size at the SDS doping concentration of 1.6 × 10^−5^ mg·mL^−1^. The enlarged pores size of the Pb(NO_3_)_2_ film help to infiltrate more MAX to conduct reactions (1) and (2). This enlarged pore size of Pb(NO_3_)_2_ film is partially supported by the slightly enhanced absorbance measurements of the Pb(NO_3_)_2_ film after SDS doping due to larger scattering, as UV-Vis spectra of Pb(NO_3_)_2_ film shown in Figure S3. However, the addition of excessive concentrations will hinder the formation of the porous Pb(NO_3_)_2_ film. Apparently, SDS promotes the nucleation and growth rate of perovskite by increasing the pores of Pb(NO_3_)_2_ film. Firstly, when Pb(NO_3_)_2_ infiltrates into the MAX solution, perovskite is rapidly formed on its surface through reactions (1) and (2). Some studies have also reported that the newly formed perovskite is in a metastable state and is easily affected by NO_3_^−^. Due to the smaller pores of the control sample, a large amount of NO_3_^−^ is trapped in the MAPbI_3_ lattice due to its slowed diffusion. NO_3_^−^ can combine with MA^+^ to cause the collapse of the metastable perovskite structure, which destroys most of the metastable perovskite. Prolonged incubation in MAX solution will gradually accumulate stable perovskite. Thus, the accumulation of stable perovskite will be pronounced for the sample with suitable pore sizes. To further verify this inference, we used a digital camera to take real-time photos to record the dynamic conversion process from Pb(NO_3_)_2_ to perovskite during the experiments, as shown in Figure 1i. Initially, the surface color of Pb(NO_3_)_2_ is almost the same before immersion in MAX solution. After 120 s of immersion, the surface of the control sample turns yellow, which can be understood as a large amount of Pb(NO_3_)_2_ being converted into PbI_2_, while the SDS-treated sample appears brown, a mixture of black and yellow. This can be explained as the generation of a large amount of perovskite in addition to PbI_2_. In four minutes, both samples turn black with the faster color change of the SDS-doped sample.

The conversion process inferred from the digital photos can be verified by XRD measurements. As shown in Figure 1j,k, both control and the SDS-treated groups show the characteristic diffraction peaks of Pb(NO_3_)_2_ initially. After 120 s immersion in MAX solution, PbI_2_ is detected at 12.64° in both groups with a weak perovskite peak at 14.27° in the SDS-treated group, indicating that the reaction rate between Pb(NO_3_)_2_ and MAX is accelerated by SDS treatment. Further immersion in MAX solution gradually decreases PbI_2_ and increases perovskite in both groups. Figure 1l calculates the proportion of MAPbI_3_ generated in each group based on the peak intensity of perovskite and the sum of perovskite and PbI_2_. According to the calculation results, the forming of perovskite in the SDS-treated group is faster and the proportion of MAPbI_3_ is always greater than that in the control group, confirming the phenomenon shown in Figure 1i.

Long-alkyl-chain surfactants are regarded as effective additives for altering the rheological properties of inks and improving the quality of perovskite film. Theoretically, low interfacial free energy is more conducive to liquid infiltration. SDS consists of a long hydrocarbon compound with a sulfonic acid functional group and a small inorganic cation (Na^+^), having a passivating effect both at grain boundaries and on crystal planes [26]. The electrostatic potential distribution is shown in Figure S4. As FTIR shows in Figure S5, the characteristic stretching vibration peak of SDS was detected at around 2900 cm^−1^ after doping SDS with Pb(NO_3_)_2_, and the position of SO_3_^-^ shifted from the original 1127 to 1150 cm^−1^, indicating the existence of SDS and its interaction with Pb(NO_3_)_2_.

In this paper, the influence of surfactants on crystal nucleation was discussed based on the classical thermodynamic theory [27]. The nucleation rate **V_1_** can be expressed as:

(3) $$V_{1}=\mathrm{kAexp}(\frac{-\Delta G^{*}}{K_{B}T}{)}^{1/2}$$

In the formula, **k** is a constant, **K_B_** is the Boltzmann constant, **T** is the absolute temperature, **A** is a complex function of the molecular-level diffusion kinetic parameters, and ΔG* is the critical free energy of nucleation. Heterogeneous nucleation sites are limited by the available surface area of substrates and colloidal particles in the precursor solution. The relationship between homogeneous and heterogeneous nucleation is expressed as:

(4) $$\Delta G_{heterogeneous\;nucleation}^{*}=\Delta G_{homogeneous\;nucleation}^{*}\;f\left(\theta \right)$$

where

(5) $$f\left(\theta \right)=\frac{2-3cos\theta +cos^{3}\theta }{4}$$

**Figure 2:**
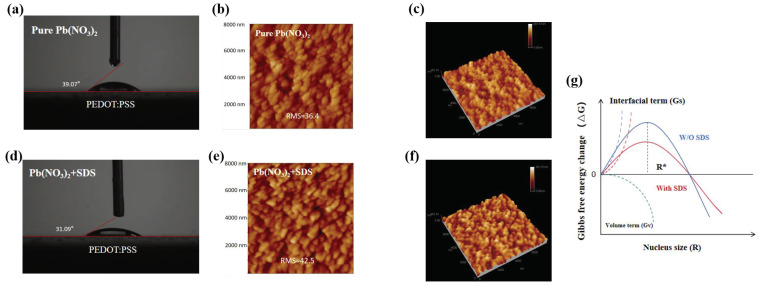
(**a**,**d**) The static contact angles of the Pb(NO_3_)_2_ solutions with and without SDS doping, respectively. (**b**,**e**) The AFM images of the Pb(NO_3_)_2_ films with and without SDS doping, respectively. (**c**,**f**) The three-dimensional AFM images of the Pb(NO_3_)_2_ films with and without SDS doping, respectively. (**g**) Gibbs free energy of perovskite nucleation with and without SDS.

According to the test results of the static contact angle in Figure 2a,d, *θ* decreases from 39.07° to 31.09° to lower the energy barrier required for nucleation, as shown in Figure 2g. The nucleation rate is thus accelerated by the reduced barrier. Figure 2b,e display the surface roughness measured by atomic force microscopy. The average surface roughness of the Pb(NO_3_)_2_ film increased from 36.4 to 42.5 nm after SDS doping. The increased surface roughness can be attributed to the formation of additional nucleation sites, which can facilitate the growth of high-quality perovskite films on the substrate [28,29].

**Figure 3:**
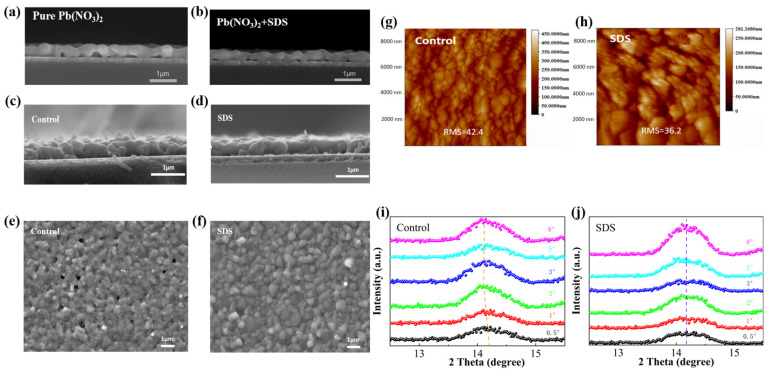
The cross-section SEM images of (**a**) Pb(NO_3_)_2_ and (**b**) Pb(NO_3_)_2_-SDS-based film; the cross-section SEM images of (**c**) control- and (**d**) SDS-based perovskite film; the SEM top view of (**e**) control- and (**f**) SDS-based perovskite film; the AFM diagram of (**g**) control- and (**h**) SDS-based perovskite film; GI-XRD spectra of (**i**) control- and (**j**) SDS-based perovskite film at different incident angles.

To investigate the impact of SDS treatment on perovskite film morphology, SEM characterizations on the cross-sections of Pb(NO_3_)_2_ films and the surfaces/cross-sections of perovskite films were conducted. Compared to the control sample without SDS doping (Figure 3a), the cross-section of Pb(NO_3_)_2_ film after SDS doping exhibits closer contact with the bottom interface (Figure 3b). Control perovskite films exhibit rugged surfaces with base pore defects due to incomplete precursor conversion, where a surface perovskite layer obstructs MAX infiltration, leaving unreacted Pb(NO_3_)_2_. In contrast, SDS-treated films feature enlarged Pb(NO_3_)_2_ pores, enabling deeper MAX penetration and more thorough conversion, yielding uniform, compact morphologies. Control films also show granular crystals from volume expansion stress and unstable decomposition products (e.g., PbI_2_) [30,31], which induce electrical shunting and recombination [32]. SDS treatment reduces these defects by increasing nucleation sites and pore roughness, promoting larger, denser crystals. AFM (Figure 3g,h) confirms smoother SDS-treated surfaces (RMS: 36.2 nm vs. 42.4 nm), enhancing contact with subsequent layers such as PC_61_BM for better carrier extraction. Additionally, SDS lowers the precursor solution’s contact angle (5.13° vs. 7.65°, as shown in Figure S6), improving wettability. The lower contact angle means smaller surface energy, which is conducive to the infiltration and crystallization of perovskite [33]. Grazing-incidence XRD can reveal residual stress in films from lattice expansion. As shown in Figure 3i,j, as the incident angle increases, the diffraction peaks of the samples in the control group shift to lower angles, indicating tensile stress due to lattice expansion [34]. However, SDS-treated films exhibit negligible stress due to accelerated conversion kinetics, yielding high-quality perovskite layers with minimized structural imperfections.

**Figure 4:**
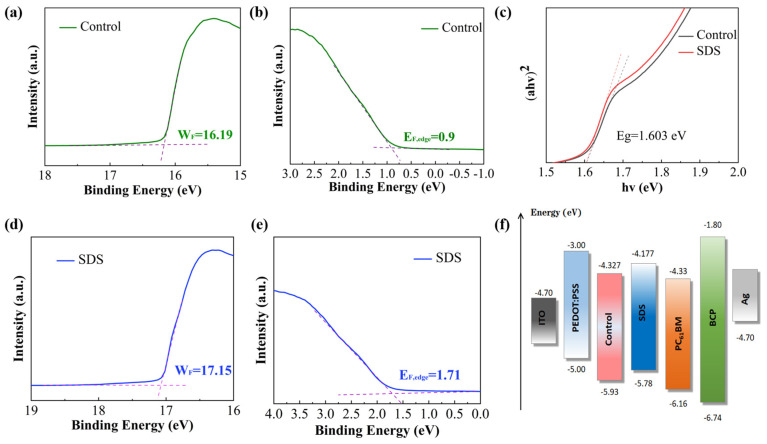
(**a**,**d**) The low energy electron cut-off edge (E_cut-off_) of perovskite samples. (**b**,**e**) The Fermi edge (E_F_,edge) of perovskite samples. (**c**) Bandgap diagram of perovskite. (**f**) Energy level diagram of different layers in the device.

UPS was employed to characterize the perovskite films of the control and SDS-treated groups, aiming to investigate the effect of SDS incorporation on interfacial hole extraction. As shown in Figure 4a,d, the low-energy electron **cut-off** edges of the control and SDS-treated perovskite films are located at **16.19** and **17.15 eV**, respectively. According to the formula E_F_ = 21.22 − E_cut-off_, the Fermi levels of the control and SDS-treated perovskite films are calculated to be **5.03** and **4.07 eV**, respectively. As depicted in Figure 4b,e the Fermi edges of the control and SDS-treated groups are located at **0.9** and **1.71 eV**, respectively. Based on the formula E_VB_ = E_F_ − E_F, edge_, the valence band energy levels (E_VB_) of the control and SDS-treated groups are calculated to be **5.93** and **5.78 eV**, respectively. To further analyze the energy level parameters, the optical absorption spectra of both films were characterized by UV-Vis spectroscopy. As shown in Figure 4c and S4, the optical bandgaps are both calculated to be **1.603 eV**. Using these data, the conduction band energy levels (E_CB_) of the control and SDS-treated films are derived to be **4.327** and **4.177 eV**, respectively. Finally, the energy level alignment diagram is summarized in Figure 4f Compared with the control group, the minimum of the conduction band in the SDS-treated perovskite film is closer to that of the PEDOT:PSS film. This reduced energy offset facilitates hole extraction from the perovskite to PEDOT:PSS, thereby minimizing open-circuit voltage (V_OC_) losses. Additionally, the low energy barrier between the perovskite and PC_61_BM enhances electron injection efficiency. The synergistic improvement in hole extraction and electron injection efficiency after SDS modification contributes to an increased fill factor (FF) of the device.

**Figure 5:**
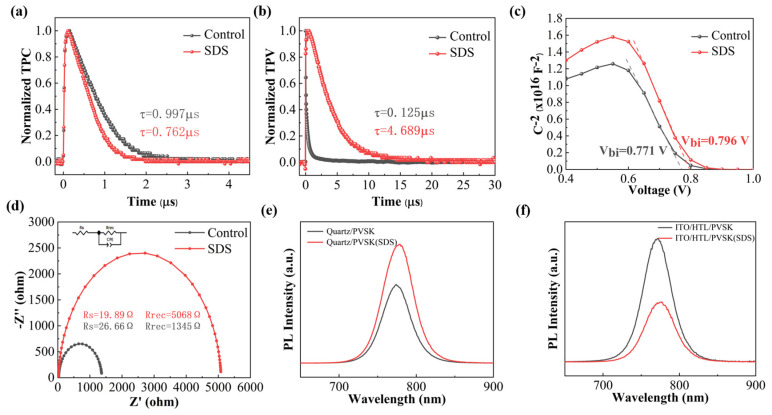
The (**a**) TPC and (**b**) TPV of control- and SDS-based devices. (**c**) The Mott–Schottky plots of the control- and SDS-based devices. (**d**) The Nyquist diagram control- and SDS-based devices at the frequency range of 3 Hz-1000 kHz under AM 1.5 G simulated sunlight. The steady state PL of (**e**) quartz- and quartz/SDS-based perovskite films, and (**f**) control- and SDS-based perovskite films.

Defect states significantly influence carrier recombination in PSCs. To investigate interfacial carrier dynamics, TPC and TPV measurements were performed. As shown in Figure 5a,b the TPC decay time of the SDS-treated device (**0.762 µs**) is shorter than that of the control group (**0.997 µs**), indicating enhanced charge extraction efficiency. Conversely, the TPV decay time of the SDS-treated device (**4.689 µs**) is longer than that of the control group (**0.125 µs**), suggesting reduced interfacial defects and suppressed non-radiative recombination. As shown in Figure 5c, Mott–Schottky measurements reveal that the flat-band potential (V_bi_) of the SDS-treated device increases by **0.025 V** compared to the control group, which directly contributes to the improved V_OC_. A higher V_bi_ enhances the driving force for charge separation and extraction [35]. The Nyquist plots of the devices in the control and the SDS-treated groups measured by EIS further verify this conclusion. Recombination resistance (R_rec_) in the low-frequency range of EIS represents the recombination resistance, indicating the non-radiative recombination at the interface. The semicircle in the high-frequency range of EIS represents the charge transfer resistance (R_s_), indicating the charge transfer and interfacial charge extraction in the device. As shown in Figure 5d, after SDS treatment, R_s_ decreases and R_rec_ increases. The smaller R_s_ is, the less the charge transfer resistance will be, which is more conducive to charge extraction. The larger R_rec_ is, the greater the resistance to non-radiative recombination will be. In addition, PL measurements (Figure 5f) demonstrate significantly stronger PL quenching in the SDS-treated perovskite film, indicative of efficient charge extraction. To exclude the influence of non-radiative recombination, control experiments were conducted on quartz substrates without charge transport layers (Figure 5e). The SDS-treated perovskite film exhibits higher PL intensity on quartz, confirming that the enhanced quenching in full devices arises from improved interfacial charge extraction rather than defect-related losses.

**Figure 6:**
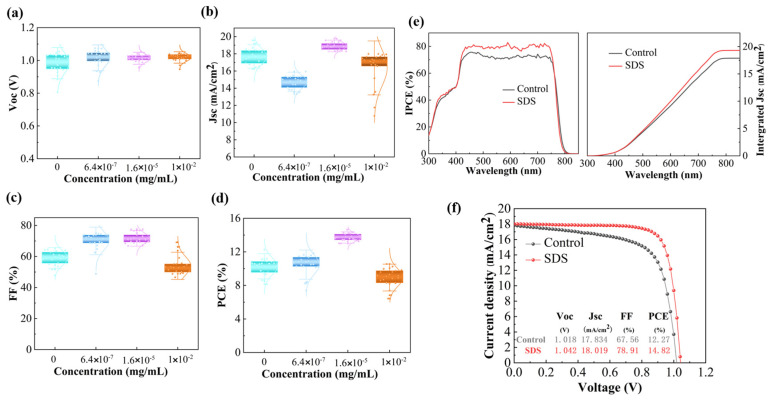
Device performance distribution with different concentrations (0, 6.4 × 10^−7^, 1.6 × 10^−5^, 1 × 10^−2^ mg·mL^−1^) of SDS from 30 cells, showing (**a**) Voc, (**b**) Jsc, (**c**) FF, and (**d**) PCE; (**e**) IPCE curves and integrated J_sc_ of the champion PSCs; (**f**) J-V curves of the champion PSCs.

We prepared PSC devices with a planar inverted structure using aqueous Pb(NO_3_)_2_ as a precursor. The statistical results of the impact of SDS doping (0, 6.4 × 10^−7^, 1.6 × 10^−5^, 1 × 10^−2^ mg·mL^−1^) on the device performance are shown in Figure 6a–d. Figure 6f presents the J-V curves of the champion devices. Compared with the control device (with a J_SC_ of **17.834 mA·cm^−2^**, a V_OC_ of **1.018 V**, an FF of **67.56%**, and a PCE of **12.27%**), the PCE of the SDS-treated device (at a concentration of 1.6 × 10^−5^ mg·mL^−1^) reaches **14.82%** (with a J_SC_ of **18.019 mA·cm^−2^**, a V_OC_ of **1.042 V**, an FF of **78.91%**, and a PCE of **14.82%**). The significant increase in V_OC_ and the improvement in FF are mainly due to the fact that, after SDS treatment, rapid nucleation and growth resulted in better perovskite film and the defects were passivated. In addition, the better energy level matching was also a contributor to the increased V_OC_. As shown in Figure 6e, the measurement of incident photon-to-current conversion efficiency (IPCE) verifies the rationality of J_SC_. The integrated current densities of the control and the SDS-treated devices were **18.12** and **18.63 mA·cm^−2^**, respectively, being consistent with the measured ones. The IPCE spectrum of the SDS-treated device was improved across the entire visible range, which can be attributed to the benefits mentioned above.

**Figure 7:**
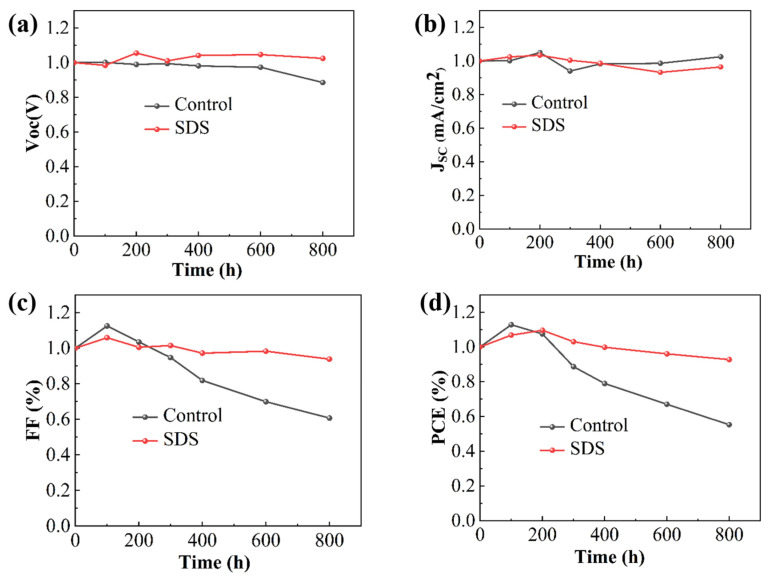
Stability tests of devices in the control and the SDS modification group (**a**) Voc, (**b**) Jsc, (**c**) FF, and (**d**) PCE.

To test the stability of the device, we placed the prepared device in a glove box filled with N_2_ for the stability test of the device, as shown in Figure 7. We tested the performance of the device for **800 h**. According to the test results in Figure 7, it can be known that the performance of the control group device only maintained about **60%** of the original after **800 h** of stability test. However, the devices modified by SDS maintained about **90%** of the original. The improvement in the stability of the devices is attributed to the perovskite films with higher quality and fewer defect states.

## 3. Experimental

### 3.1. Materials

Lead (II) nitrate (Pb(NO_3_)_2_, 99.99%) and isopropanol (IPA, 99.8%) were purchased from Aladdin (Shanghai, China). PEDOT:PSS (Heraeus Clevios AI 4083, 1.3–1.7% in H_2_O dispersion), methylammonium iodide (MAI, 99.5%), methylammonium chloride (MACl, 99.5%), -phenyl C61-butyric acid methyl ester (PC61BM, 99.5%), and bathocuproine (BCP, 99.0%) were obtained from Xi’an Yuri Solar Co., Ltd. (Xi’an, China). Chlorobenzene (CB, 99.8%, ultra-dry) was sourced from MERYER (Shanghai, China). Indium tin oxide (ITO) substrates with a sheet resistance of ~15 Ω/□ were procured from Advanced Election Technology Co., Ltd. (Shenzhen, China).

### 3.2. Device Fabrication

The ITO glass substrates were sequentially cleaned through the following procedure: first, ultrasonic cleaning with detergent at 55 °C for 1 h using a KQ3200DV cleaner (Shumei, Kunshan, China), followed by at least three cycles of ultrasonic rinsing with deionized water (10 min per cycle), and finally dried under a dry N_2_ stream. A PEDOT:PSS solution (V_PEDOT:PSS_:V_water_ = 1:6) was spin-coated onto the ITO substrates at 6000 rpm for 40 s after 1 min of static deposition, followed by thermal annealing at 150 °C for 30 min. The substrates were then transferred into a custom-built glove box maintained at 20% relative humidity (RH).

Perovskite films were fabricated using a sequential deposition method. First, an aqueous Pb(NO_3_)_2_ solution (1.8 M) was spin-coated onto the PEDOT:PSS layer at 4000 rpm for 30 s, followed by immediate annealing at 100 °C for 10 min. Subsequently, the Pb(NO_3_)_2_ films were subjected to two cycles of MAX solution immersion (15 mg·mL^−1^ in IPA, W_MAI_:W_MACl_ = 4:1) to form the control perovskite films. Notably, the films were rinsed with IPA after each immersion to remove by-products. For SDS-treated devices, SDS solutions of varying concentrations (1, 0.1, and 0.001 mg·mL^−1^ in deionized water) were used as the solvent while maintaining the Pb(NO_3_)_2_ concentration at 1.8 M. All other processing steps remained identical to those of the control films. Following the immersion process, the films were annealed at 100 °C for 30 min. The device fabrication continued with spin-coating of PC61BM (20 mg·mL^−1^ in CB) at 3000 rpm for 30 s, followed by annealing at 90 °C for 30 min. A BCP layer (0.5 mg·mL^−1^ in IPA) was then deposited by spin-coating at 2000 rpm for 30 s. Finally, Ag cathodes (~120 nm) were thermally evaporated under a vacuum of 1 × 10^−4^ Pa, completing the planar inverted PSCs with the structure of ITO/PEDOT:PSS /Perovskite/PC61BM/BCP/Ag. The fabricated devices had an effective area of 0.0625 cm^2^.

### 3.3. Characterizations

The J-V curves and steady-state output of the devices were gained by a source meter (Keithley, 2400, Cleveland, OH, USA) under the simulated light (AM 1.5G, 100 mW·cm^−2^) from a Newport simulator (94043A; Irvine, CA, USA). The EQE was tested by calculating the photocurrent and light intensity, measured using a lock-in amplifier (SR-830; Stanford Research Systems, Sunnyvale, CA, USA) by focusing a monochromatic light beam onto devices and a calibrated silicon detector, respectively. The phase and crystallinity of the films were tested by X-ray diffraction (XRD, Shimadzu, 7000, Kyoto, Japan) with a Cu Kα radiation source under 40 kV and 30 mA. Electrochemical impedance spectra (EIS) were measured with the electrochemical workstation (Chenhua, 660D, Shanghai, China) on a probe station (TTPX, Lakeshore, Westerville, OH, USA). The morphology of the perovskite film was characterized by scanning electron microscopy (SEM, JSM-6700F, JEOL Ltd., Akishima, Tokyo, Japan) at an accelerating voltage of 10 kV. For the test of transient photovoltage (TPV) and transient photocurrent (TPC), a pulsed laser with a pulse width of 6 ns and a wavelength of 532 nm was used as the excitation light source, and the Agilent DSO-X 3102A oscilloscope (Santa Clara, CA, USA) with a bandwidth of 1 GHz was adopted to collect the decays of voltage and current. UV-Vis absorption spectra in the range of 300–900 nm were obtained with the commercial spectrophotometer (Shimadzu UV-2550). The energy levels were measured by ultraviolet photoelectron spectroscopy (UPS, Shimadzu, AXIS SUPRA^+^, Japan). Surface roughness was quantified using atomic force microscopy (AFM) with the CSPM 5500 system. Contact angles (CA) were diligently measured using a contact angle goniometer (YIKE-360A, Chengde Precision Test Instrument Factory, Chengde, China). The FTIR spectra were recorded with a Spectrum Two 20A10370 of PE (PerkinElmer, Waltham, MA, USA). An FLS1000 instrument from Edinburgh Instruments (Livingston Village, UK) achieved steady-state photoluminescence (PL).

## 4. Conclusions

We present an effective additive strategy for fabricating high-performance planar inverted aqueous perovskite solar cells (PSCs) through SDS incorporation in Pb(NO_3_)_2_ precursor solutions. The SDS modification induces critical surface property alterations in Pb(NO_3_)_2_ films, which (1) enhance MAX diffusion kinetics through optimized interfacial interactions, (2) promote reaction dynamics for accelerated perovskite conversion, (3) increase surface nucleation density for improved crystal growth orientation, and (4) generate comprehensive defect passivation through stress relaxation and energy level realignment. These synergistic effects collectively reduce interfacial and bulk defect densities compared to control devices, suppress non-radiative recombination pathways, and enhance carrier extraction efficiency. The optimized devices achieve a champion power conversion efficiency of **14.82%**, representing a **20.8%** enhancement over control devices (**12.27%**).

## Data Availability

Data will be made available on request.

## Supplementary Materials

The following supporting information can be downloaded at: https://www.mdpi.com/article/10.3390/molecules30102146/s1, Figure S1: Structural formula of sodium dodecyl sulfonate (SDS); Figure S2: Preparation process of the device with two times of MAX incubations; Figure S3: Absorption spectra of Pb(NO_3_)_2_ and Pb(NO_3_)_2_-SDS at doping concentration of 1.6 × 10^−5^ mg·mL^−1^; Figure S4: Electrostatic potential distribution map of SDS; Figure S5: (a) FTIR spectra of pristine Pb(NO_3_)_2_, SDS and Pb(NO_3_)_2_-SDS grinding mixture. (b) An enlargement of (a); Figure S6: Contact angle measurements of the MAX solution on the surface of Pb(NO_3_)_2_ (left) and Pb(NO_3_)_2_-SDS (right).
