# Supplementary material for: Controlling Crystallization of Aqueous-Processed Planar Perovskite Films via Sodium Dodecyl Sulfonate Surfactant Modulation
Source: Molecules. 2025 May 13;30(10):2146. doi: 10.3390/molecules30102146 (PMC12114574; doi:10.3390/molecules30102146)
Supplement: Supplementary file 1 [file molecules-30-02146-s001.zip › molecules-3564114-supplementary.pdf]

SUPPORTING INFORMATION

Controlling Crystallization of Aqueous-Processed Planar Perovskite Films via Sodium Dodecyl Sulfonate Surfactant Modulation

Na Zheng, Cunyun Xu, Xiaofeng He, Gaobo Xu, Jiancheng You, Zhongjun Dai, Han Jiang, Qianqian Zhang and Qunliang Song *

Institute for Clean Energy and Advanced Materials, School of Materials and Energy, Southwest University, Chongqing 400715, China; zn1981608813@email.swu.edu.cn (N.Z.); cyxuamos@foxmail.com (C.X.); hexf1992@163.com (X.H.); gbxu1805@163.com (G.X.); youjiancheng2021@163.com (J.Y.); jlovendai@163.com (Z.D.); 18283003178@163.com (H.J.); 18311651723@163.com (Q.Z.)
* Correspondence: qlsong@swu.edu.cn

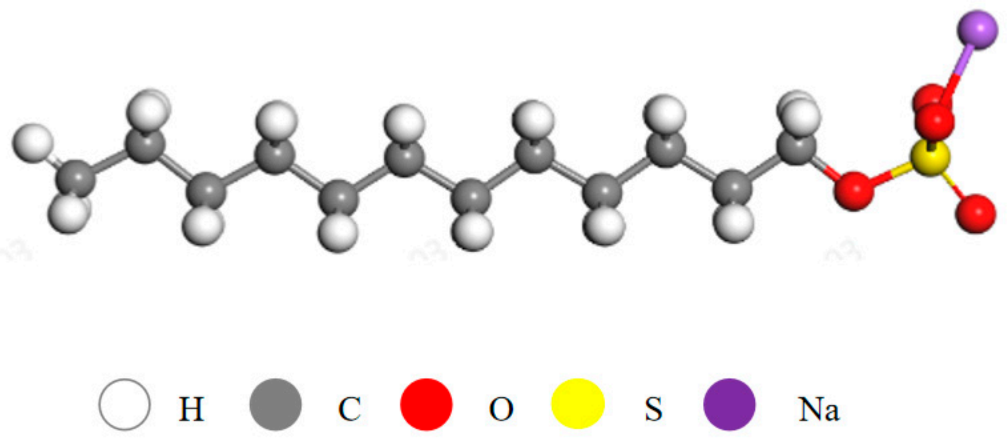

Fig. S1. Structural formula of sodium dodecyl sulfonate (SDS).

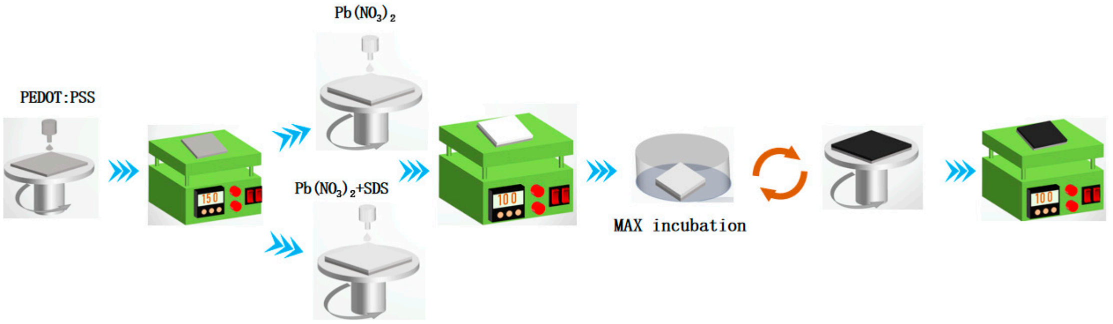

Fig. S2. Preparation process of the device with two times of MAX incubations.

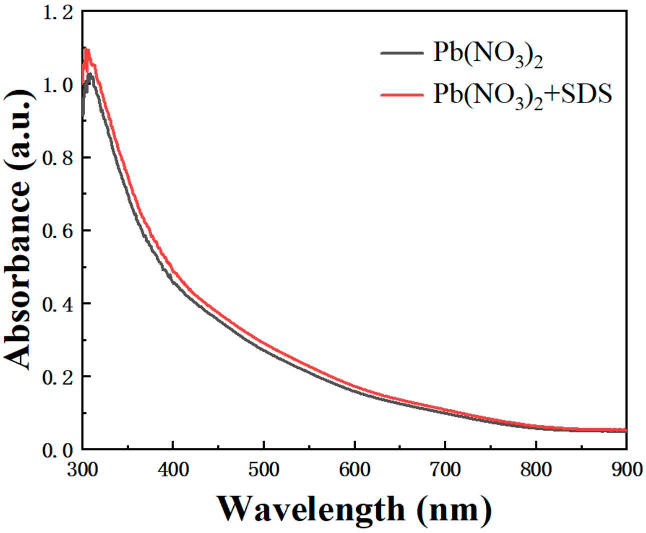

Fig. S3. Absorption spectra of $Pb(NO_3)_2$ and $Pb(NO_3)_2$-SDS at doping concentration of 1.6×10^{-5} mg·mL^{-1}.

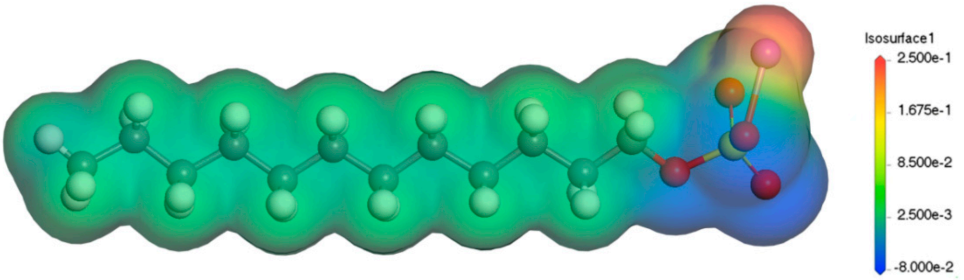

Fig. S4. Electrostatic potential distribution map of SDS.

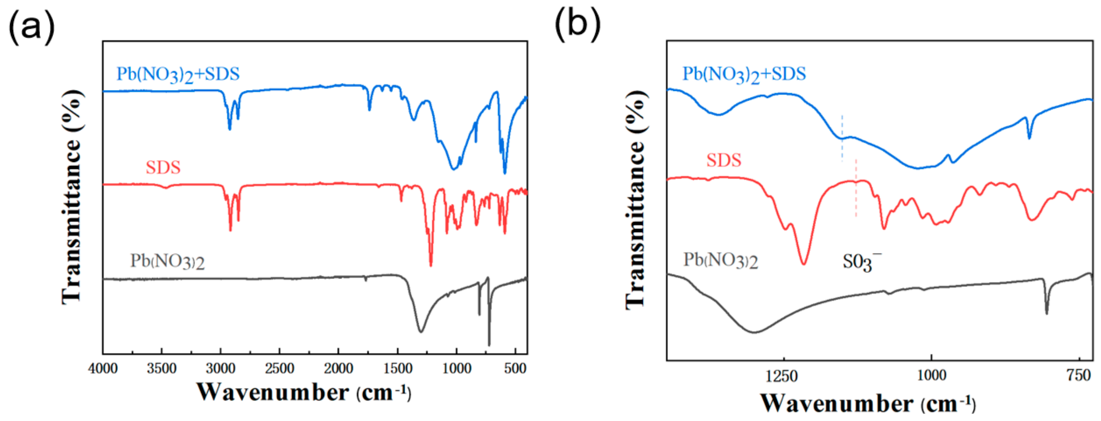

Fig. S5. (a) FTIR spectra of pristine $Pb(NO_3)_2$, SDS and $Pb(NO_3)_2$-SDS grinding mixture. (b) An enlargement of (a).

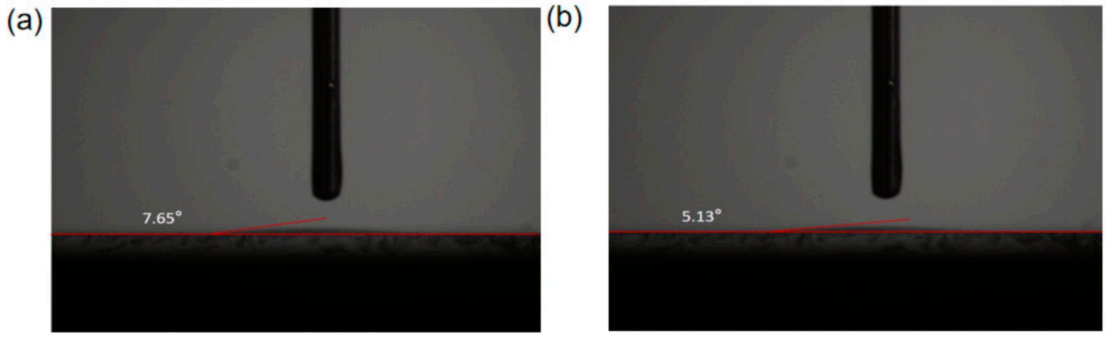

Fig. S6. Contact angle measurements of the MAX solution on the surface of $Pb(NO_3)_2$ (left) and $Pb(NO_3)_2$-SDS (right).
